# Supplementary material for: Measurement of Serum IgG Anti-Integrin αvβ6 Autoantibodies Is a Promising Tool in the Diagnosis of Ulcerative Colitis
Source: J Clin Med. 2022 Mar 28;11(7):1881. doi: 10.3390/jcm11071881 (PMC8999661; doi:10.3390/jcm11071881)
Supplement: Supplementary file 1 [file jcm-11-01881-s001.zip › Supplementary Figure S1.pdf]

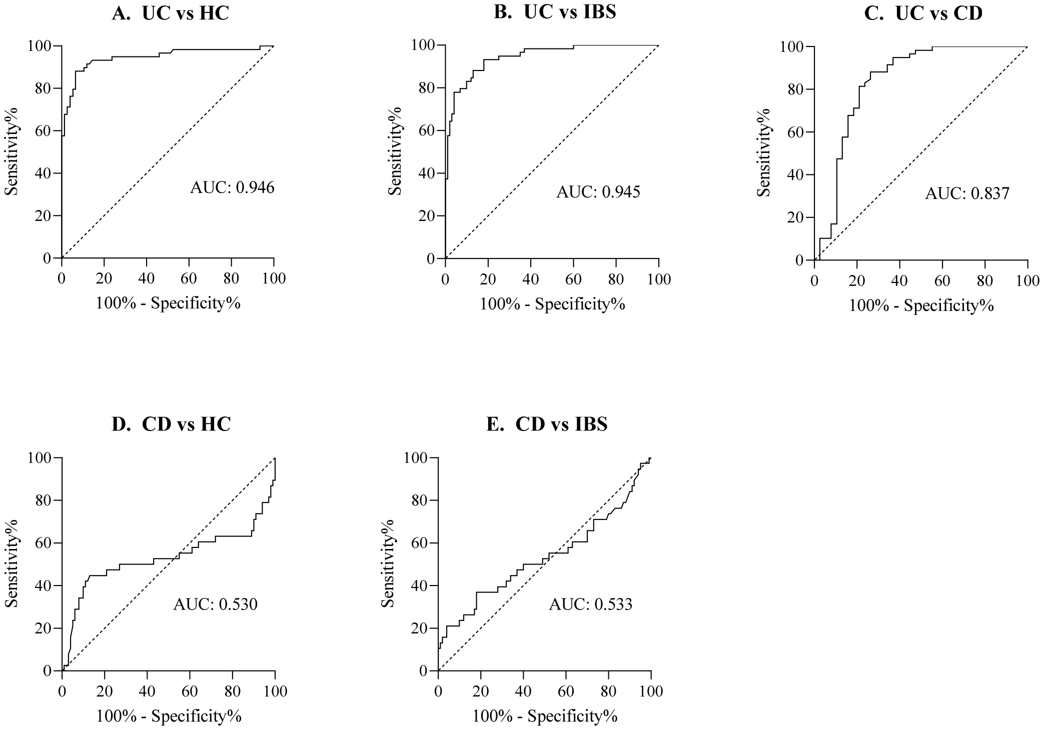

Supplementary Figure S1. Receiver operating characteristic (ROC) curves for IgG anti-αvβ6 integrin autoantibodies.
