# Supplementary material for: Measurement of Serum IgG Anti-Integrin αvβ6 Autoantibodies Is a Promising Tool in the Diagnosis of Ulcerative Colitis
Source: J Clin Med. 2022 Mar 28;11(7):1881. doi: 10.3390/jcm11071881 (PMC8999661; doi:10.3390/jcm11071881)
Supplement: Supplementary file 1 [file jcm-11-01881-s001.zip › Supplementary Table S1.pdf]

Supplementary Table S1. UC and CD patient characteristics

Sample	Age (yr)	Sex	CRP (mg/dL)	fCP (mg/kg)	*Partial Mayo score	*Harvey-Bradshaw index	Treatment
UC 1	42	F	2.25	6.7	1	n/a	Prednisolone. Mesalazine
UC 2	25	F	2.32	32.5	0	n/a	Mesalazine
UC 3	34	F	1.79	6.9	0	n/a	Azathioprine
UC 4	48	F	1.79	29.0	1	n/a	Olsalazin
UC 5	49	M	1.47	21.9	0	n/a	No medicaton
UC 6	73	F	5.97	3607.4	5	n/a	Sulfasalazine
UC 7	25	M	2.68	2771.1	1	n/a	Mesalazine
UC 8	31	M	1.95	3.5	0	n/a	Vedolizumab
UC 9	60	F	1.76	14.3	1	n/a	Infliximab. Mercaptopurine
UC 10	40	F	3.18	10.5	0	n/a	Prednisolone
UC 11	28	F	1.81	8.2	7	n/a	Mesalazine
UC 12	50	F	2.22	323.6	7	n/a	No medication
UC 13	24	M	6.76	12.9	6	n/a	Prednisolone. Sulfasalazine Mesalazine suppository
UC 14	42	M	1.68	-	0	n/a	Infliximab. Mesalazine Mercaptopurine
UC 15	25	F	2.07	680.2	1	n/a	Azathioprine Mesalazine
UC 16	72	M	6.34	394.2	1	n/a	Sulfasalazine
UC 17	28	F	2.58	4206.7	5	n/a	Azathioprine. Vedolizumab
UC 18	34	F	2.48	1418.6	5	n/a	Prednisolone. Azathioprine
UC 19	65	M	3.64	-	1	n/a	Sulfasalazine
UC 20	50	M	2.00	509.4	1	n/a	No medication
UC 21	21	M	10.49	1666.8	6	n/a	Prednisolone. Mesalazine
UC 22	47	M	18.19	3828.6	6	n/a	Mesalazine
UC 23	34	M	12.22	-	3	n/a	No medication
UC 24	24	F	11.49	-	3	n/a	Azathioprine
UC 25	71	F	2.41	921.3	0	n/a	Mesalazine
UC 26	27	M	2.80	2679.7	7	n/a	Mesalazine
UC 27	35	F	2.30	-	7	n/a	No medication
UC 28	56	F	12.01	1674.5	6	n/a	No medication
UC 29	56	M	1.81	312.5	5	n/a	No medication
UC 30	23	M	4.42	161.6	6	n/a	Mesalazine. Mesalazine suppository
UC 31	25	M	2.25	207.2	6	n/a	No medication
UC 32	79	M	3.18	618.1	2	n/a	Prednisolone. Mesalazine

UC 33	38	M	14.02	-	6	n/a	Prednisolone Mesalazine
UC 34	54	M	7.08	101.0	4	n/a	Sulfasalazine Azathioprine
UC 35	47	F	2.12	63.0	6	n/a	Mesalazine. Infliximab
UC 36	63	F	2.05	749.7	3	n/a	Prednisolone Vedolizumab Tioguanine
UC 37	21	M	1.90	-	2	n/a	Sulfasalazine
UC 38	56	M	8.62	-	5	n/a	Mesalazine
UC 39	26	M	3.36	-	6	n/a	No medication
UC 40	55	F	17.18	299.0	4	n/a	Prednisolone
UC 41	24	M	1.88	2852.2	0	n/a	Mesalazine
UC 42	46	F	1.77	3.0	0	n/a	Mesalazine
UC 43	54	F	2.16	61.4	4	n/a	Olsalazine
UC 44	30	M	5.17	851.6	8	n/a	Mesalazine. Mesalazine suppository Prednisolone Azathioprine
UC 45	18	F	1.78	24.0	3	n/a	Mesalazine
UC 46	28	F	2.06	1253.5	4	n/a	Mesalazine
UC 47	46	F	2.62	1862.3	3	n/a	Sulfasalazine
UC 48	26	M	1.81	76.7	3	n/a	Mesalazine
UC 49	38	M	1.81	691.1	6	n/a	Mesalazine Mesalazine suppository Prednisolone suppository
UC 50	60	M	1.84	27.6	3	n/a	Mesalazine
UC 51	50	M	2.25	45.5	1	n/a	Mesalazine
UC 52	28	M	3.25	378.8	1	n/a	Mesalazine
UC 53	43	F	1.68	135.0	3	n/a	Prednisolone suppository
UC 54	33	M	2.33	610.9	6	n/a	Mesalazine Prednisolone suppository
UC 55	38	M	3.90	1817.0	7	n/a	Mesalazine
UC 56	55	F	2.08		0	n/a	Mercaptopurine
UC 57	37	M	1.58	130.9	0	n/a	Mesalazine Azathioprine Prednisolone Prednisolone suppository
UC 58	21	M	1.84	-	1	n/a	Mesalazine Mercaptopurine Infliximab
UC 59	30	M	1.86	173.3	0	n/a	Vedolizumab Prednisolone. Mesalazine

CD 1	38	M	5.01	37.0	n/a	10	Prednisolone
CD 2	56	M	4.43	2694.8	n/a	4	Prednisolone
CD 3	64	M	4.33	18.2	n/a	5	Prednisolone
CD 4	23	M	2.50	167.2	n/a	14	No medication
CD 5	57	F	2.02	119.9	n/a	5	Azathioprine
CD 6	34	M	2.60	338.2	n/a	1	Azathioprine. Prednisolone
CD 7	25	M	2.02	31.9	n/a	1	Mesalazine. Azathioprine. Infliximab
CD 8	60	F	2.33	-	n/a	1	Infliximab. Methotrexate
CD 9	34	F	137.01	40.1	n/a	13	No medication
CD 19	23	M	1.89	440.5	n/a	6	No medication
CD 11	37	M	17.06	1754.1	n/a	6	Mesalazine
CD 12	67	M	2.92	989.8	n/a	3	Mesalazine. Azathioprine
CD 13	64	F	1.68	109.5	n/a	5	Prednisolone
CD 14	39	F	25.04	1203.6	n/a	26	Azathioprine. Adalimumab
CD 15	33	F	4.35	14028.1	n/a	7	No medication
CD 16	23	F	3.14	30.7	n/a	4	Infliximab. Azathioprine
CD 17	31	F	2.09	-	n/a	8	Infliximab. Budenoside
CD 18	65	F	3.14	333.5	n/a	3	No medication
CD 19	30	F	3.07		n/a	15	Infliximab
CD 20	20	M	2.12	14.4	n/a	3	Infliximab. Azathioprine
CD 21	74	M	3.88	535.7	n/a	2	Sulfasalazine
CD 22	29	F	1.74	5537.4	n/a	6	Azathioprine
CD 23	51	F	2.14	1516.2	n/a	8	Azathioprine
CD 24	59	F	2.23	704.8	n/a	5	No medication
CD 25	78	F	3.16	69.3	n/a	2	Mercaptopurine
CD 26	28	F	10.82	-	n/a	6	No medication
CD 27	24	M	2.58	-	n/a	4	Mesalazine. Azathioprine
CD 28	67	M	1.40	51.3	n/a	4	No medication
CD 29	71	M	6.52	958.0	n/a	3	Vedolizumab. Budenoside. Sulfasalazine
CD 30	32	M	2.19	76.4	n/a	4	Mercaptopurine. Infliximab
CD 31	27	F	1.95	62.3	n/a	10	Budenoside. Mercaptopurine. Adalimumab
CD 32	70	F	1.84	112.5	n/a	2	Budenoside

CD 33	32	F	2.53	-	n/a	4	Mesalazine
CD 34	63	M	2.22	165.8	n/a	1	No medication
CD 35	64	F	1.60	369.2	n/a	7	No medication
CD 36	44	F	2.47	9.6	n/a	3	Azathioprine
CD 37	60	M	1.97	49.5	n/a	12	Infliximab.Mesalazin. Azathioprine
CD 38	28	M	4.14	-	n/a	2	Infliximab

UC. ulcerative colitis; CD. Crohn’s disease

* Disease activity: UC (Partial Mayo Scoring Index): < 2 Remission. 2-4 mild. 5-6 moderate. > 6 severe. CD (Harvey-Bradshaw Index): < 5 remission. 5-7 mild. 8-16 moderate. > 16 severe.
