# Supplementary material for: Measurement of Serum IgG Anti-Integrin αvβ6 Autoantibodies Is a Promising Tool in the Diagnosis of Ulcerative Colitis
Source: J Clin Med. 2022 Mar 28;11(7):1881. doi: 10.3390/jcm11071881 (PMC8999661; doi:10.3390/jcm11071881)
Supplement: Supplementary file 1 [file jcm-11-01881-s001.zip › Supplementary Table S2.pdf]

Supplementary Table S2. IBS patient characteristics

Sample	Age (yr)	Sex	CRP (mg/dL)	fCP (mg/kg)	*IBS-Symptom Severity Score
IBS 1	43	F	2.19	213.07	341
IBS 2	53	F	1.87	9.88	341
IBS 3	59	F	1.76	37.10	400
IBS 4	18	M	2.51	34.42	146
IBS 5	23	M	2.07	108.64	335
IBS 6	34	F	6.61	4.64	212
IBS 7	40	M	3.11	28.83	402.5
IBS 8	20	F	2.35	8.93	258
IBS 9	26	F	1.62	30.16	236.5
IBS 10	59	M	2.21	29.40	387.5
IBS 11	28	F	1.87	6.99	325
IBS 12	65	F	2.08	53.45	243
IBS 13	50	F	1.93	83.35	263.5
IBS 14	21	M	1.75	19.06	425
IBS 15	28	M	1.81	-	354
IBS 16	51	F	2.15	8.29	450
IBS 17	54	F	2.1	17.65	325
IBS 18	31	F	1.57	-	323
IBS 19	49	M	1.79	8.51	370
IBS 20	39	F	1.94	6.65	302
IBS 21	36	F	1.98	523.89	346
IBS 22	23	F	5.84	55.65	317.5
IBS 23	26	M	1.7	7.42	260
IBS 24	36	F	1.67	12.55	370
IBS 25	23	F	2.56	21.43	310
IBS 26	50	F	3.22	8.98	159
IBS 27	23	F	1.65	6.43	372.5
IBS 28	63	F	2.26	12.52	355.5
IBS 29	44	F	2.57	6.40	278
IBS 30	34	F	1.98	6.49	417.5
IBS 31	31	F	1.99	24.30	465
IBS 32	26	M	1.97	4.85	210
IBS 33	58	F	2.19	89.52	242.5
IBS 34	32	F	2.12	7.42	356
IBS 35	34	F	1.65	8.42	495
IBS 36	34	F	2.07	27.30	215
IBS 37	51	F	1.8	5.64	265
IBS 38	36	M	1.69	-	406
IBS 39	31	F	3.01	-	450
IBS 40	38	F	3.94	11.00	325
IBS 41	32	F	2.55	5.06	224
IBS 42	53	M	1.99	88.63	232
IBS 43	42	F	4.06	496.46	117
IBS 44	21	M	2.08	66.17	330
IBS 45	27	F	1.63	3.39	240
IBS 46	51	F	2.12	8.74	286
IBS 47	63	F	2.51	15.38	435
IBS 48	49	F	2.68	8.05	379

IBS 49	28	F	2.17	20.17	149
IBS 50	57	F	1.51	164.46	385
IBS 51	31	F	1.71	31.43	257.5
IBS 52	27	F	2.07	88.39	326
IBS 53	28	M	15.91	15.38	358
IBS 54	63	M	2.36	4.00	280
IBS 55	37	F	4.59	64.58	282
IBS 56	32	F	1.7	-	413
IBS 57	60	F	1.68	16.51	248.5
IBS 58	64	F	2.33	2.36	99
IBS 59	25	F	25.83	8.22	299
IBS 60	35	M	2.03	3.10	482
IBS 61	61	F	1.97	36.70	157
IBS 62	58	M	2.56	9.70	189
IBS 63	24	F	1.65	9.82	323
IBS 64	25	F	1.92	26.43	267
IBS 65	75	F	2.59	232.97	290
IBS 66	55	M	10.73	41.31	238
IBS 67	55	F	1.74	45.45	240
IBS 68	45	F	1.97	17.25	320
IBS 69	56	M	1.94	19.93	264
IBS 70	43	F	3.18	25.84	181
IBS 71	21	F	1.95	148.14	368
IBS 72	30	F	1.97	20.14	252
IBS 73	30	F	2.82	-	395
IBS 74	44	M	3.08	-	322
IBS 75	37	F	1.69	4.01	274
IBS 76	35	F	2.03	40.48	219
IBS 77	73	M	1.77	9.91	251
IBS 78	70	F	2.17	20.00	283
IBS 79	64	M	2.88	180.52	373
IBS 80	31	F	1.86	382.57	332
IBS 81	39	F	1.7	-	214
IBS 82	32	F	2.22	27.76	401
IBS 83	31	M	2.68	-	353
IBS 84	48	F	1.43	11.42	297
IBS 85	36	F	2.16	184.89	395
IBS 86	35	F	2.03	11.02	375
IBS 87	31	M	1.68	12.80	331
IBS 88	45	F	2.63	8.03	396
IBS 89	31	F	1.94	5.46	354
IBS 90	39	M	1.87	11.62	305
IBS 91	58	F	2.53	13.84	246
IBS 92	42	M	3.39	17.97	347
IBS 93	32	M	1.74	3.88	320
IBS 94	26	M	3.13	156.43	311
IBS 95	59	F	1.48	10.01	273
IBS 96	46	M	1.78	9.88	273
IBS 97	29	F	1.57	57.82	249
IBS 98	29	M	2.54	12.11	270
IBS 99	29	F	1.65	13.68	351
IBS 100	56	F	4.77	51.07	373

IBS: Inflammatory Bowel Syndrome.

* IBS symptom severity score: < 175 mild, 175-300 moderate, >300 severe.
