# Supplementary material for: Measurement of Serum IgG Anti-Integrin αvβ6 Autoantibodies Is a Promising Tool in the Diagnosis of Ulcerative Colitis
Source: J Clin Med. 2022 Mar 28;11(7):1881. doi: 10.3390/jcm11071881 (PMC8999661; doi:10.3390/jcm11071881)
Supplement: Supplementary file 1 [file jcm-11-01881-s001.zip › Supplementary Table S3.pdf]

Supplementary Table S3. Diagnostic performance of fCP in diagnosis of IBD with the entities UC and CD.

	Sens.	Spec.	PPV	NPV	OR (95% CI)	LR	p-value #
**fCP (compliance group)**							
UC vs CD	0.708	0.258	0.597	0.364	0.845 (0.292-2.38)	0.955	0.802
UC vs IBS	0.708	0.769	0.618	0.833	8.10 (3.54-18.0)	3.07	<0.0001
CD vs IBS	0.742	0.769	0.523	0.897	9.58 (3.77-24.5)	3.22	<0.0001
IBD vs IBS	0.722	0.769	0.731	0.761	8.64 (4.29-16.8)	3.13	<0.0001
**fCP (whole study group)1)**							
UC vs CD	0.576	0.395	0.597	0.375	0.887 (0.383-1.97)	0.952	0.835
UC vs IBS	0.576	0.790	0.618	0.760	5.12 (2.58-10.1)	2.74	<0.0001
CD vs IBS	0.605	0.790	0.523	0.840	5.77 (2.54-13.0)	2.88	<0.0001
IBD vs IBS	0.588	0.790	0.731	0.664	5.36 (2.85-9.78)	2.80	<0.0001

IBD, inflammatory bowel disease; UC, ulcerative colitis; CD, Crohn's disease; IBS, irritable bowel syndrome, fCP, fecal calprotectin; Sens., sensitivity; Spec., specificity; PPV, positive predictive value; NPV, negative predictive value; OR, odds ratio; CI, confidence interval; LR, likelihood ratio.

[1)] Diagnostic performance when including the whole study group, i.e. also the patients that failed to provide stool samples, which was 12 (19.3%) for UC, 6 (16.2%) for CD and 9 (9.0%) for IBS.

[#] p-value: Fisher's exact test.
